# Supplementary material for: Modeling glioblastoma heterogeneity as a dynamic network of cell states
Source: Mol Syst Biol. 2021 Sep 16;17(9):e10105. doi: 10.15252/msb.202010105 (PMC8444284; doi:10.15252/msb.202010105)
Supplement: Supplementary file 6 — Source Data for Figure 5 [file MSB-17-e10105-s004.zip › Figure5A_sourcedata/GSEA_3017/hallmarks_stateF.GseaPreranked.1621934628066/HALLMARK_TNFA_SIGNALING_VIA_NFKB.html]

Details for gene set HALLMARK\_TNFA\_SIGNALING\_VIA\_NFKB[GSEA]

|  || Dataset | state33017 |
| Phenotype | NoPhenotypeAvailable |
| Upregulated in class | na\_pos |
| GeneSet | HALLMARK\_TNFA\_SIGNALING\_VIA\_NFKB |
| Enrichment Score (ES) | 0.49948585 |
| Normalized Enrichment Score (NES) | 2.6275587 |
| Nominal p-value | 0.0 |
| FDR q-value | 0.0 |
| FWER p-Value | 0.0 |
Table: GSEA Results Summary

  

Fig 1: Enrichment plot: HALLMARK\_TNFA\_SIGNALING\_VIA\_NFKB      
 Profile of the Running ES Score & Positions of GeneSet Members on the Rank Ordered List

  

| PROBE | GENE SYMBOL | GENE\_TITLE | RANK IN GENE LIST | RANK METRIC SCORE | RUNNING ES | CORE ENRICHMENT || 1 | CDKN1A |  |  | 4 | 2.089 | 0.0701 | Yes |
| 2 | CCL2 |  |  | 7 | 1.774 | 0.1303 | Yes |
| 3 | PNRC1 |  |  | 23 | 1.070 | 0.1603 | Yes |
| 4 | PTX3 |  |  | 25 | 1.061 | 0.1964 | Yes |
| 5 | F3 |  |  | 41 | 0.978 | 0.2233 | Yes |
| 6 | SAT1 |  |  | 45 | 0.957 | 0.2548 | Yes |
| 7 | ABCA1 |  |  | 48 | 0.938 | 0.2862 | Yes |
| 8 | SQSTM1 |  |  | 54 | 0.914 | 0.3154 | Yes |
| 9 | PLK2 |  |  | 77 | 0.805 | 0.3331 | Yes |
| 10 | BTG2 |  |  | 124 | 0.708 | 0.3366 | Yes |
| 11 | NAMPT |  |  | 168 | 0.634 | 0.3389 | Yes |
| 12 | ATP2B1 |  |  | 170 | 0.632 | 0.3602 | Yes |
| 13 | NINJ1 |  |  | 182 | 0.610 | 0.3762 | Yes |
| 14 | CEBPB |  |  | 190 | 0.601 | 0.3937 | Yes |
| 15 | CD44 |  |  | 191 | 0.600 | 0.4144 | Yes |
| 16 | BTG1 |  |  | 222 | 0.574 | 0.4205 | Yes |
| 17 | PDLIM5 |  |  | 223 | 0.572 | 0.4402 | Yes |
| 18 | SLC2A3 |  |  | 228 | 0.569 | 0.4579 | Yes |
| 19 | CEBPD |  |  | 234 | 0.566 | 0.4751 | Yes |
| 20 | HES1 |  |  | 320 | 0.501 | 0.4538 | Yes |
| 21 | IL6ST |  |  | 321 | 0.500 | 0.4710 | Yes |
| 22 | CSF1 |  |  | 333 | 0.495 | 0.4830 | Yes |
| 23 | ID2 |  |  | 366 | 0.483 | 0.4851 | Yes |
| 24 | TIPARP |  |  | 472 | 0.444 | 0.4527 | Yes |
| 25 | TSC22D1 |  |  | 473 | 0.443 | 0.4680 | Yes |
| 26 | DRAM1 |  |  | 519 | 0.427 | 0.4622 | Yes |
| 27 | CFLAR |  |  | 569 | 0.408 | 0.4540 | Yes |
| 28 | FOS |  |  | 592 | 0.401 | 0.4578 | Yes |
| 29 | IRS2 |  |  | 595 | 0.400 | 0.4707 | Yes |
| 30 | IER5 |  |  | 605 | 0.396 | 0.4802 | Yes |
| 31 | TNC |  |  | 613 | 0.393 | 0.4906 | Yes |
| 32 | IFIT2 |  |  | 624 | 0.391 | 0.4995 | Yes |
| 33 | NFE2L2 |  |  | 663 | 0.381 | 0.4953 | No |
| 34 | SNN |  |  | 732 | 0.363 | 0.4770 | No |
| 35 | VEGFA |  |  | 840 | 0.339 | 0.4401 | No |
| 36 | SOCS3 |  |  | 869 | 0.332 | 0.4388 | No |
| 37 | BHLHE40 |  |  | 928 | 0.321 | 0.4235 | No |
| 38 | GADD45A |  |  | 1028 | 0.305 | 0.3890 | No |
| 39 | KDM6B |  |  | 1139 | 0.286 | 0.3489 | No |
| 40 | RNF19B |  |  | 1148 | 0.285 | 0.3551 | No |
| 41 | NFAT5 |  |  | 1198 | 0.279 | 0.3424 | No |
| 42 | PLPP3 |  |  | 1213 | 0.277 | 0.3456 | No |
| 43 | PTPRE |  |  | 1243 | 0.275 | 0.3419 | No |
| 44 | SGK1 |  |  | 1283 | 0.270 | 0.3335 | No |
| 45 | TAP1 |  |  | 1317 | 0.266 | 0.3277 | No |
| 46 | GEM |  |  | 1391 | 0.257 | 0.3034 | No |
| 47 | PDE4B |  |  | 1404 | 0.256 | 0.3068 | No |
| 48 | JUNB |  |  | 1466 | 0.251 | 0.2877 | No |
| 49 | HBEGF |  |  | 1583 | -0.282 | 0.2447 | No |
| 50 | PHLDA1 |  |  | 1718 | -0.324 | 0.1950 | No |
| 51 | IER2 |  |  | 2034 | -0.524 | 0.0700 | No |
| 52 | SERPINE1 |  |  | 2161 | -0.843 | 0.0418 | No |
Table: GSEA details [plain text format]

  

Fig 2: HALLMARK\_TNFA\_SIGNALING\_VIA\_NFKB: Random ES distribution      
 Gene set null distribution of ES for **HALLMARK\_TNFA\_SIGNALING\_VIA\_NFKB**
